# Maxillary Sinus Augmentation with Xenogenic Collagen-Retained Heterologous Cortico-Cancellous Bone: A 3-Year Follow-Up Randomized Controlled Trial

**DOI:** 10.3390/dj12020033

**Published:** 2024-02-03

**Authors:** Francisco Correia, Sónia Gouveia, António Campos Felino, Ricardo Faria-Almeida, Daniel H. Pozza

**Affiliations:** 1 Specialization in Periodontology and Implants, Faculty of Dental Medicine, University of Porto, 4200-393 Porto, Portugal; franciscodcorreia@gmail.com (F.C.); rfaperio@gmail.com (R.F.-A.); 2 Department of Electronics, Telecommunications and Informatics (DETI), Institute of Electronics and Informatics Engineering of Aveiro (IEETA), University of Aveiro, 3810-193 Aveiro, Portugal; sonia.gouveia@ua.pt; 3 Intelligent Systems Associate Laboratory (LASI), Portugal, 4800-058 Guimarães, Portugal; 4 Department of Oral Surgery and Periodontology, Faculty of Dental Medicine, University of Porto, 4200-393 Porto, Portugal; antonioccfelino@gmail.com; 5 Associated Laboratory for Green Chemistry (LAQV) of the Network of Chemistry and Technology (REQUIMTE), 4050-342 Porto, Portugal; 6 Experimental Biology Unit, Department of Biomedicine, Faculty of Medicine of Porto, University of Porto, 4200-319 Porto, Portugal; 7 Institute for Research and Innovation in Health and IBMC (i3S), University of Porto, 4200-135 Porto, Portugal

**Keywords:** sinus augmentation, xenografts, bone transplantation, dental implants, graft survival, patient preferences

## Abstract

Sinus augmentation procedures have become a valuable solution for patients with posterior maxillary edentulism. The objective of this study was to explore the efficacy and safety of porcine xenograft with collagen supplementation as a potential alternative to autologous bone grafts in lateral sinus augmentation over a three-year follow-up period. Twelve patients, each with bilateral posterior maxillary edentulism, were enrolled and randomly allocated to receive either a porcine xenograft or an autologous graft. Comprehensive assessments, including clinical and radiographic evaluations, were conducted at specific intervals, including implant stability, marginal bone loss, prosthetic and biological complications, and patient preferences. The results demonstrated no significant differences between the two graft materials in terms of implant survival, marginal bone loss, and patient preferences after three years of follow-up. Only one implant was affected by peri-implantitis, and prosthesis-related complications were present in one patient possibly due to bruxism. In conclusion, these findings suggest that a porcine xenograft with collagen supplementation may be a viable alternative to an autograft in lateral sinus augmentation procedures. The high implant survival rate, minimal complications, and patient satisfaction indicate the potential clinical relevance of this graft material and should be further investigated to confirm these promising results.

## 1. Introduction

The lateral sinus lift, initially described in the 1980s, has become one of the main surgical techniques in dental implantology [1,2]. This approach allows for bone regeneration in the posterior maxilla and is the technique with the most scientific documentation out of all sinus lift procedures [3,4,5]. A significant milestone in the evolution of this technique was marked in 1988 when Wood and Moore introduced the concept of utilizing intra-oral autologous bone grafts in lateral sinus augmentation [6]. Even today, many experts still consider autologous bone grafts to be the gold standard in this domain, primarily due to their osteogenic, osteoinductive, and osteoconductive properties [7,8,9,10,11].

In contemporary clinical practice, there is a growing emphasis on patient-centered care, along with an increased focus on assessing patient-reported outcome measures (PROMS). The drive for less invasive procedures has led to a search for alternatives aiming to match or surpass autologous bone grafts, not only in efficacy but also in mitigating patient morbidity, minimizing the required surgical procedures, and saving time. However, autologous bone grafts present limitations such as the requirement for a second surgical area and a rapid, unpredictable absorption rate, which may necessitate additional procedures and could potentially compromise the long-term stability of the graft material [8,12,13].

The literature describes various alternatives to autologous bone grafts, such as autografts, allografts, xenografts, and alloplastic materials, along with different sources and combinations. These have been used in lateral sinus lift procedures, yet it is important to note that not all grafts exhibit the same pattern of osteoinduction and osteoconduction [8,14,15,16,17,18]. Consequently, there are different results in terms of new bone formation and the quantity of residual graft material that remains in the grafted area [14,15]. Thus, there is still a lot of controversy surrounding the selection of the ideal graft to be used [8], which justifies well-conducted studies in this field of dentistry.

The cortico-medullary porcine xenograft Osteobiol Mp3^®^ by Tecnoss^TM^, Italy, exhibits a granulometry ranging from 600 to 1000 μm and is distinguished by the incorporation of a gel containing 10% collagen of type I and III within the matrix. This xenograft is conveniently provided in prepared syringes, streamlining the manipulation process [9,19].

In a previous histological analysis, carried out six months after clinical sinus lift surgery, which compared the porcine xenograft with a 10% collagen to the intra-oral autologous bone graft, no differences were observed between the two graft materials [20]. The question that remains is whether there is a difference between these graft materials in implant survival, marginal bone loss, and other clinical parameters over time. To the best of our knowledge, there is no other randomized clinical trial aimed at determining whether there is a difference in long-term implant survival rate when using a porcine xenograft with 10% collagen.

The objective of this randomized split-mouth controlled clinical trial was to assess whether there is a difference in terms of implant survival rate, implant marginal bone loss, clinical complications, patient preferences, and prosthetic complications after a 3-year period between the use of a porcine xenograft with 10% collagen and an intra-oral autologous bone graft for lateral sinus augmentation.

## 2. Materials and Methods

### 2.1. Study Design

In this clinical trial, the blind randomized split-mouth design (RCT) was implemented, aligning our approach with the principles outlined in the CONSORT Statement and the ethical guidelines set forth in the World Medical Association Declaration of Helsinki [21]. This study had been previously evaluated and approved by the Ethics and Research Committee of the Faculty of Dentistry at the University of Porto, Portugal, under protocol number 00977.

Additionally, this study’s protocol is registered at trial.gov under reference NCT01836744.

Twelve consecutive patients were enrolled in this randomized split-mouth clinical trial, with the follow-up period lasting from March 2013 to June 2017, comprising 1 year of treatment and 3 years of follow-up. Twenty-four maxillary sinuses were divided into two groups: one group received autologous grafts, while the other group received porcine xenografts with 10% collagen, as previously described in a prior publication [20].

The primary advantage of a split-mouth design is that both materials are tested within the same patient, which allows patients to be their own controls (i.e., removes the intra-patient variation from the analysis). This typically increases the statistical power (i.e., the probability of rejecting a false null hypothesis) of the analysis. Our choice for a sample size of 12/24 (#patients/#sinus) followed previous bilateral trials comparing two materials, including 11/22 [22], 8/16 [23], and a multicenter study with an 11/22 [24] sample size. Within Osteobiol MP3^®^-based research, clinical trials with a single graft per patient are commonly used either to compare two materials, e.g., 18/18 [25] and 20/20 [26], or to assess the performance of the xenograft material itself, e.g., 10/10 [27].

For the random allocation of a graft material (autologous graft or xenograft) to each regenerated sinus, a software-based randomization method was used on www.randomizer.org. The outcomes of this randomized allocation were securely enclosed within opaque, sealed envelopes, and these envelopes were exclusively opened by the surgeon following the elevation of both sinus Schneider membranes. Importantly, the allocation scheme remained undisclosed to clinical examiners and patients throughout the study.

Participants eligible for this study had to meet specific inclusion criteria, which encompassed being 18 years of age or older, possessing the ability to comprehend and provide informed consent, and presenting with bilateral posterior maxillary edentulism characterized by a comparable degree of bone resorption (vertical bone height falling within the range of 1–5 mm as assessed using computed tomography—CT). In contrast, the exclusion criteria included individuals who had undergone radiation therapy in the head and neck, those with immunosuppression or immunocompromised status, patients receiving intravenous bisphosphonate treatment, individuals with untreated periodontitis, those who showed oral hygiene and motivation, uncontrolled diabetes mellitus, pregnant or breastfeeding individuals, those with psychiatric problems, and those with untreated sinusitis [28].

### 2.2. Treatment Procedures

The preoperative preparation for each patient began prior to the surgery, including dental polishing ten days before, administering 2 g of amoxicillin one hour prior to the procedure, and using a chlorhexidine 0.2% mouthwash one minute before starting the sinus lift.

**Figure 1:**
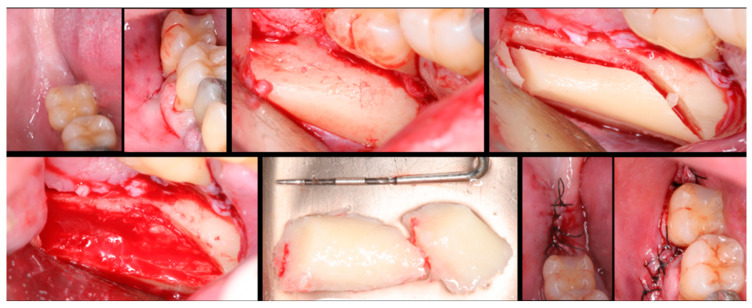
Surgical steps for harvesting autologous bone graft from the mandibular branch. In the first row, left to right: mandibular ramus, incision, flap detachment, and graft drilling. In the second row, left to right: graft removal, graft measurement, and sutures.

**Figure 2:**
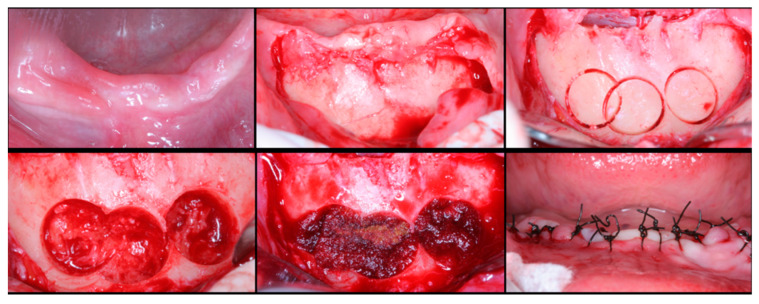
Surgical steps for harvesting autologous bone graft from the mental symphysis. In the first row, left to right: anterior mandibular overview, incision and flap detachment, graft drilling. In the second row, left to right: graft removal, chin filling, and sutures.

**Figure 3:**
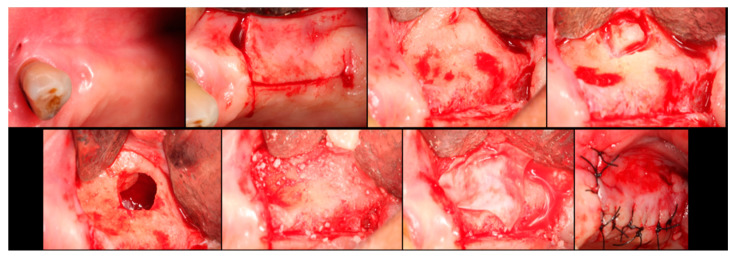
Step-by-step surgical demonstration of maxillary sinus elevation with xenograft. In the first row, left to right: incision in the fourth quadrant, flap detachment, and lateral osteotomy. In the second row, left to right: accessing the sinus, filling with xenograft, collagen membrane positioning, and sutures.

**Figure 4:**
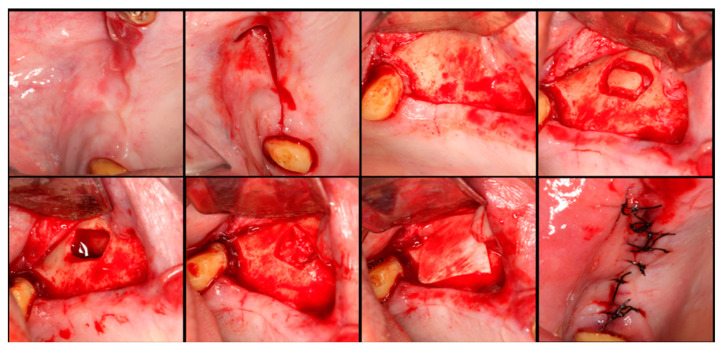
Step-by-step surgical demonstration of maxillary sinus elevation with autologous bone graft. In the first row, left to right: incision in the fourth quadrant, flap detachment, and lateral osteotomy. In the second row, left to right: accessing the sinus, filling with autograft, collagen membrane positioning, and sutures.

The surgical process, performed by the same surgeon, involved local anesthesia followed by incisions, flap detachment, osteotomy, and membrane elevation. Autologous bone from the mandibular branch (Figure 1) or the mental symphysis (Figure 2), or a porcine xenograft (Figure 3) were used as the graft materials for sinus augmentation. The protocol for this study involved using either two or three syringes of Oteobiol MP3, delivering a volume of approximately 2 to 3 cc per sinus. The contralateral sinus received a similar amount of autologous bone, either from the mental region (desmal with probably some bone of enchondral origin) or the mandibular ramus (desmal origin). Figure 3 and Figure 4 depict one example from each side of maxillary sinus augmentation treatment. Post surgery, the patients followed a prescribed protocol. After six months, a follow-up CT scan was conducted, and dental implants were placed. The final phase involved inserting healing abutments or prostheses, ensuring that no tooth–implant connections were established [20].

### 2.3. Follow-up

The evaluations included maintenance, prosthesis examination, and occlusion adjustments every 6 months after the prosthetic loading was performed. Clinical and radiographic evaluations were performed in the first and third years after implant loading. These evaluations included patient preferences, the assessment of implant stability, and marginal bone loss as well as the identification of any prosthetic or biological complications. Implants that did not achieve osteointegration were identified in cases involving implant mobility, infection, or the need for implant removal. Prosthesis failure was characterized as the inability to achieve the planned oral rehabilitation.

**Figure 5:**
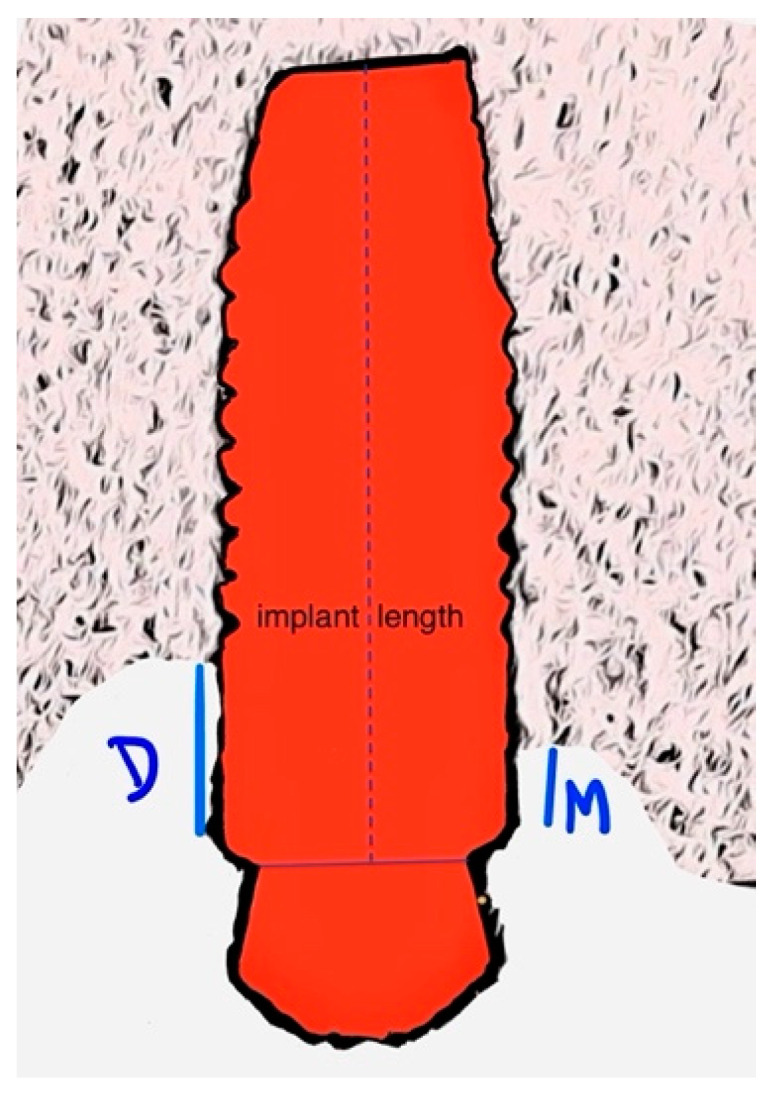
Illustrative image of how marginal bone loss is measured. D—distal; M—mesial.

To monitor changes in the peri-implant marginal bone level, periapical radiographs were taken with the paralleling technique at the initial prosthetic loading and at the one- and three-years-post-implant function. These radiographs were used to measure the distance between the marginal bone level and the implant/abutment junction at both mesial and distal sites (Figure 5). Using the Scion Image software (Scion Corporation, Frederick, MD, USA), each image was individually calibrated based on the corresponding implant length. Precise measurements of the mesial and distal bone crest levels adjacent to each implant were recorded with a precision of 0.01 mm, with reference points established at the coronal margin of the implant collar and the most coronal point of bone–implant contact [29].

### 2.4. Statistical Analysis 

Data organization and descriptive statistics were initially conducted using Microsoft Excel^TM^ version 16.10 and IBM-SPSS^TM^ version 25.0, developed by IBM Corp. in Armonk, NY, USA. 

Conventional parametric and non-parametric statistical tests at a 5% significance level were performed. The statistical comparisons were based on paired *t*-tests (parametric) and Wilcoxon signed-rank tests (non-parametric), with the assumption of normality being evaluated through the Shapiro–Wilk test. The results reported in this research are based on the parametric approach for statistical inference as there was no statistical evidence against the normality of the populations underlying the analysis. Furthermore, parametric ANOVA was used to estimate and test the time effect on radiographic bone loss as well as the time vs. material interaction effects.

## 3. Results

### 3.1. Patient and Intervention Characteristics 

**Figure 6:**
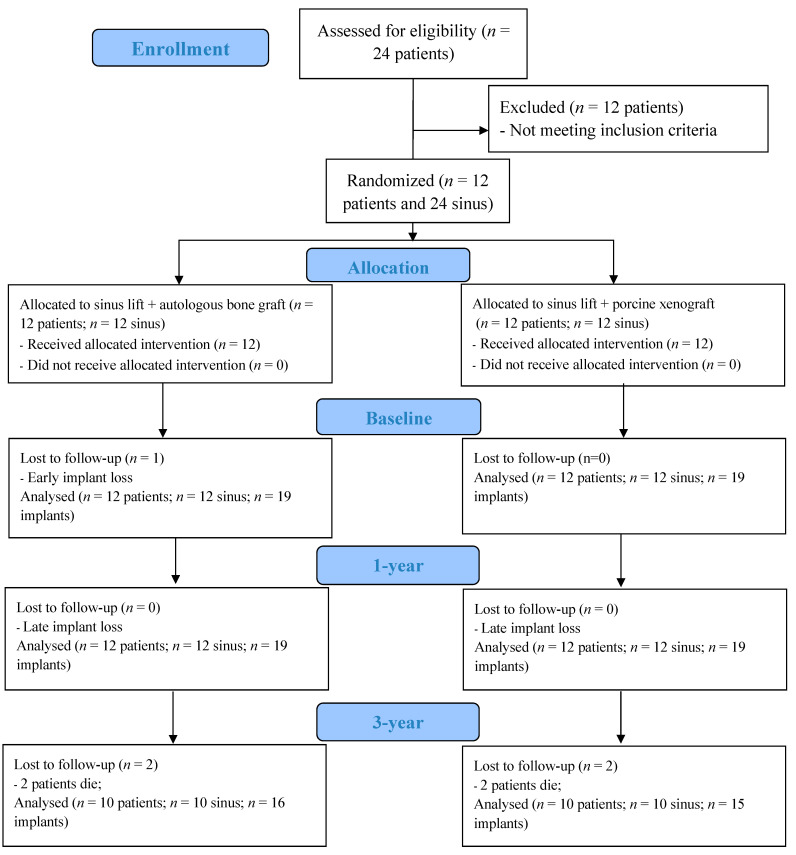
CONSORT—flow diagram.

Twenty-four patients were initially enrolled in the study, from which a sample of twelve patients meeting the inclusion criteria was selected (Figure 6). The patient sample consisted of six females and six males, with an average age of 59.7 ± 8.7 years. In terms of smoking habits, at the beginning of the randomized clinical trial, six patients were non-smokers, three were light smokers (≤10 cigarettes/day), one was a heavy smoker (≥11 cigarettes/day), and two were former smokers. Most of the patients had up to one pathology (9 out of 12) and were taking at least one prescribed medication (11 out of 12).

The autologous bone graft was harvested from the mandibular branch in 83.3% of the cases and from the chin in the remaining 16.7% of the cases. Following the protocol, after 6 months, a total of 39 dental implants were placed, with 16 implants of 9 mm in length and 23 implants of 11 mm in length.

Between the 1-year and 3-year follow-up, unfortunately, two patients passed away, one due to a heart attack and the other due to breast cancer. One of the patients experienced a heart attack but made a full recovery without any lasting effects, while another suffered a stroke resulting in some motor impairments. Additionally, one patient developed pulmonary emphysema and subsequently quit smoking. It is important to note that all these medical complications are being managed by the patients’ healthcare providers, and the individuals are closely monitored in terms of their medication and treatment. We understand that these health problems do not appear to be directly related to bone regeneration or implant treatment.

By the third year of treatment, when the patients were asked about their treatment preferences, all the patients reported that either “Neither” or “Both procedures were equally good”. This indicates that there was no strong preference for one sinus lift procedure over the other.

### 3.2. Implant Survival, Biological Complication, and Marginal Implant Bone Loss

Out of the 39 implants that were placed, one of them did not achieve osteointegration at the xenograft site. However, no more implants were lost during the 3-year follow-up period, resulting in an implant survival rate of 95% for the xenograft side and 100% for the autologous graft side.

Regarding biological complications, in the third year, one implant on the autologous graft side was diagnosed with peri-implantitis. This implant was successfully treated with a combination of non-surgical and regenerative surgical treatment.

A radiographic analysis of marginal bone loss was conducted, excluding the implants of patients who dropped out and the implant affected by peri-implantitis, as their inclusion could introduce bias into the results.

In Table 1, the variation in marginal bone loss for the 15 implants analyzed in each group between the time of prosthesis delivery and the 1-year and 3-year follow-up is reported. It is evident that, in the mesial and distal sites, there is a statistically significant marginal bone loss over time (mesial *p* = 0.001 and distal *p* = 0.001). However, when comparing the marginal bone loss over time between the two materials, it did not reach statistically significant differences (interaction effect: mesial *p* = 0.985 and distal *p* = 0.540). These results suggest that the radiographic marginal bone loss is similar for both materials.

**Figure 7:**
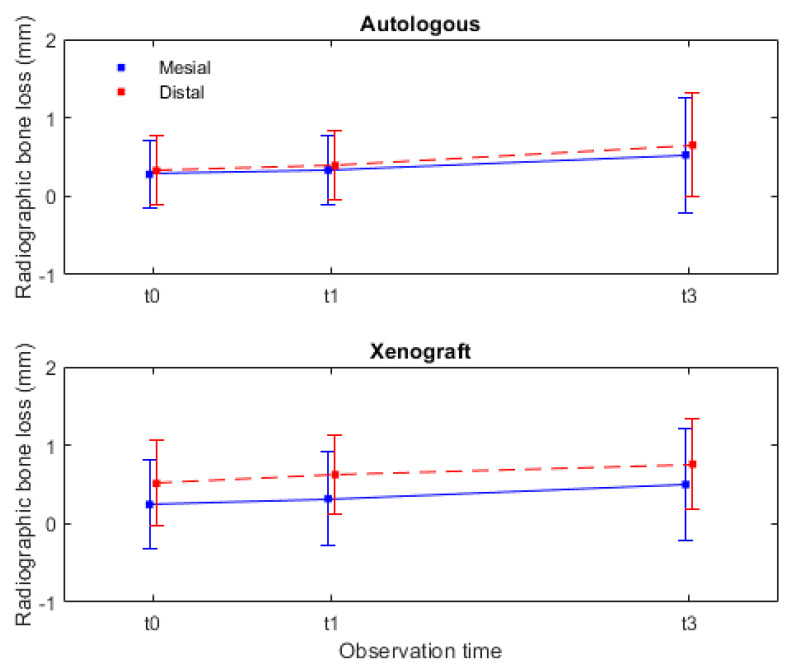
Radiographic marginal bone changes, during the 3-year observation period, assessed for both mesial and distal sites. Legend: autologous-autologous bone graft (control group); xenograft-porcine xenograft (test group); t0-initial; t1-1 year; and t3-3 years.

In Figure 7, the radiographic marginal bone loss data are presented not as an aggregate but rather divided between mesial and distal measurements. The mesial marginal bone loss for the autologous group was 0.53 ± 0.73 mm, and, for the xenograft group, it was 0.50 ± 0.71 mm. In the distal measurements, the autologous group had a bone loss of 0.65 ± 0.66 mm, while the xenograft group had a bone loss of 0.76 ± 0.58 mm. It can be statistically inferred with these data, contained in (Table 1) and Figure 7 (graphic perspective), that the radiographic marginal bone loss after 3 years was slightly higher in the distal than in the mesial sites.

### 3.3. Prosthesis Survival and Hardware Complication

In the present study, the 12 patients were categorized as follows: 2 patients received individual crowns on each augmented side, while 10 patients received crowns which were either splinted or part of a bridge. The prosthesis survival rate for the 10 patients was 100% after 3 years.

Regarding complications related to the prostheses, only one patient encountered multiple major complications, including fractures in both the right and left ceramic bridges as well as 16 screw fractures and fractures in one multi-unit component on each side. In the second year, one of the patients experienced a minor complication, which was the loosening of the crown screw in both quadrants.

## 4. Discussion

In this study, the efficacy and safety of porcine xenograft with 10% collagen compared to autograft in lateral sinus augmentation procedures was clinically evaluated. The findings over a 3-year follow-up period provided valuable insights into the clinical outcomes and complications associated with these two graft materials.

One key observation from this study is the absence of major differences in the various analyzed outcomes between the control group (autograft) and the test group (porcine xenograft with 10% collagen). This suggests that both the xenograft material with collagen supplementation and the autograft present good properties in terms of implant survival and radiographic marginal bone loss, being comparable to other previous clinical studies [30,31,32]. This finding is encouraging and highlights the potential of porcine xenograft as a viable alternative for sinus augmentation procedures, with equal or better results than other grafts, such bone substitutes alone (xenografts, synthetic alloplastic graft), autogenous bone and bone substitutes, autogenous bone particulate, and/or autogenous bone block [30,32,33].

From a clinical perspective, the porcine xenograft with 10% collagen is easy to manipulate and has good osteoconduction, as demonstrated in histologic studies [7,9,34], similar to the autologous bone but with reduced morbidity [14,35]. Furthermore, avoiding harvesting autologous bones allows for a reduction in surgical time, consequently lowering the risks associated with the procedures themselves.

The high implant survival rate observed during the follow-up period in both graft groups is reassuring and supports the use of porcine xenografts with collagen in clinical practice like previous studies with the same 3-year follow-up evaluation period [32,33]. Furthermore, the slight but similar radiographic marginal bone loss in both groups indicates that both materials can maintain the stability of the implant over the studied time. The stability in the first few years is crucial to ensure the long-term future survival of dental implant procedures. 

The radiography bone loss observed in our study is in line with the results of a systematic review and meta-analysis [32] that estimates 0.99 mm with a 95% confidence interval of 0.62–1.37 mm, demonstrating excellent implant/bone stability and biocompatibility. Minor statistical differences (less than 0.2 mm) between mesial and distal measurements can be considered not clinically relevant. Similar findings have been reported in another previous study [36], and these differences may potentially be attributed to minimal radiographic distortion. From a clinical perspective, it is crucial to conduct an ongoing analysis over time to determine whether these slight differences in radiographic marginal bone loss between groups will increase or remain stable. However, if this difference increases proportionally with time, it could be considered clinically significant, since continuous marginal bone loss could expose implant threads, facilitating peri-implantitis [37]. 

Biological complications, such as peri-implantitis, are of significant concern in implant dentistry [38]. The occurrence of peri-implantitis in only one implant in our study is a promising result, suggesting that both graft materials, when properly used, are associated with low rates of biological complications. The peri-implantitis of the implant was successfully treated with a combination, in a first stage, of non-surgical treatment and, in a second stage, with receptive surgical treatment in combination with implantoplasty. This treatment was chosen since the application of implantoplasty in a surgical approach can lead to a better clinical outcome [39]. It is noteworthy that a regenerative approach can result in radiographic bone gain; however, it is essential to consider that not all cases warrant the indication for a regenerative approach [40]. In the clinical cases that do not have clinical indications for a regenerative approach, the receptive approach is the better option and can be successfully treated as demonstrated in our patient.

A systematic review with a meta-analysis analyzed 22 manuscripts from 16 randomized trials examining decontamination methods. They all improved clinical parameters, with no single method proving to be definitively superior. Systemic antibiotics showed short-term benefits in treatment success [41]. Furthermore, antibiotics in the treatment of peri-implant diseases, particularly peri-implantitis, were shown to yield favorable clinical results for up to 12 months after therapy [42].

Preventing peri-implantitis is of utmost importance [37]. Certain factors such as a previous history of periodontitis [43], having less than 2 mm of keratinized mucosa [44], patient hygiene, smoking, including electronic cigarettes, and the use of cemented restorations [45,46,47] can elevate the risk of peri-implantitis. In our study, all the patients presented one or more of these risk factors. However, it is noteworthy that only one patient developed peri-implantitis, and this individual had both a smoking habit and a history of periodontitis.

The expected prevalence of peri-implantitis was found to be 19.53% (95% CI 12.87–26.19) at the patient-level and 12.53% (95% CI 11.67–13.39) at the implant-level, and it displayed significant variability, even after adopting a more specific clinical case definition [48]. It is well known that supportive maintenance therapy, correct implant placement, and prosthesis design (no plaque accumulation) can drastically reduce peri-implantitis occurrence [37,38,49,50]. In this context, the 6-month supportive maintenance therapy employed in our patients can be one of the key factors for the low incidence of peri-implantitis in the present study.

Prosthesis-related complications were limited to one patient. These complications are likely associated with individual factors, such as bruxism and the absence of a nightguard, rather than the choice of graft material. This emphasizes the importance of patient-specific factors that must be unveiled prior to the treatment to plan adequate post-operative care to improve implant survival and reduce the number of prosthodontic/mechanical complications [51,52].

Despite the relatively restrictive inclusion/exclusion criteria, the patients in our study represent daily clinical practice in cases of bilateral maxillary edentulism, offering a viable treatment option for individuals with low morbidity, including a consideration of factors such as age. Therefore, the choice of graft material can be influenced by various clinical factors, such as the availability of autologous bone, clinical preferences, and patient-specific characteristics.

The present study, while providing valuable insights into the efficacy and safety of porcine xenografts with collagen supplementation in lateral sinus augmentation procedures, is subject to certain limitations. First, the presence of various patient factors, such as age, overall health, and smoking habits, introduces heterogeneity that, despite the inclusion and exclusion criteria, was not completely controlled for, restricting the generalizability of our findings in the general population. The three-year follow-up of this study, though illuminating clinical outcomes, may not fully capture the long-term performance and potential complications of the entire treatment. On the other hand, longer follow-ups are difficult to perform due to dropouts. Randomization safeguards against hidden biases by creating a balanced distribution of known and unknown variables when assigning side/material to groups. Despite obtaining non-significant results on both sides, randomization could prevent the dentist from biasing the choice of a suitable side for either graft. However, in this specific context, and as a post-study verification, the immediate apparent added value of randomization may be limited. Recognizing and addressing these limitations is essential to interpret our findings accurately and guide future research aiming to improve our understanding of porcine xenograft use in sinus augmentation procedures.

In summary, the findings of this study suggest that porcine xenografts with 10% collagen may be a suitable alternative to autografts in lateral sinus augmentation procedures. Clinicians should consider adopting this xenograft material for lateral sinus augmentation procedures as it demonstrates similar clinical and radiographic results to autologous grafts, while offering an advantage in terms of reduced patient morbidity and surgical bone graft collection. Further research with larger sample sizes and longer follow-up periods may provide additional insights into the long-term performance of these graft materials.

## 5. Conclusions

After 3 years of follow-up, no major differences were observed in the various analyzed outcomes between the autograft (control group) and the porcine xenograft with 10% collagen (test group).

Throughout this follow-up period, a high implant survival rate was observed, along with a minor but comparable degree of radiographic marginal bone loss in both grafted groups. Only one biological complication (peri-implantitis) was observed in one of the implants in a patient with a history of periodontitis who also happened to be a smoker. 

Major prosthesis complications were observed in only one patient, and these complications are likely related to their bruxism and the absence of a nightguard.

## Figures and Tables

**Table 1:** Radiographic bone losses in millimeters were compared over time for mesial (M0, M1, and M3) and distal (D0, D1 and D3) sites and between autologous graft and xenograft groups.

		M0		M1		M3	
		Mean	SD	Mean	SD	Mean	SD
Graft	A	0.28	0.43	0.33	0.45	0.53	0.73
	X	0.25	0.56	0.32	0.59	0.50	0.71
	Total	0.26	0.49	0.33	0.52	0.51	0.71
		D0		D1		D3	
		Mean	SD	Mean	SD	Mean	SD
Graft	A	0.33	0.44	0.39	0.44	0.65	0.66
	X	0.51	0.55	0.63	0.50	0.76	0.58
	Total	0.42	0.50	0.51	0.48	0.71	0.61

A-autologous graft; X-xenograft groups. These comparisons display the mean and standard deviation (SD).

## Data Availability

Data are available from the corresponding author upon reasonable request.
